# Draft Genome Sequences of *Shewanella* sp. Strain UCD-FRSP16_17 and Nine *Vibrio* Strains Isolated from Abalone Feces

**DOI:** 10.1128/genomeA.00977-16

**Published:** 2016-09-15

**Authors:** Ashley Vater, Vivian Agbonavbare, Dylan A. Carlin, Griselda M. Carruthers, Adam Chac, Ladan Doroud, Samuel J. Farris, Melanya Gudzeva, Guillaume Jospin, John A. Kintner, Jonathon P. Knauss, Yi Lor, Randi Pechacek, Eden S. Rohner, Sierra M. V. Simmons, Mayya Verescshagina, Christian S. Wirawan, Leonel Zagal, David A. Coil

**Affiliations:** a Integrative Pathobiology, University of California Davis, Davis, California, USA; b University of California Davis, Davis, California, USA; c University of California Davis Genome Center, Davis, California, USA

## Abstract

We present here the draft genome sequences for nine strains of *Vibrio* (*V. cyclitrophicus*, *V. splendidus*, *V. tasmaniensis*, and three unidentified) and one *Shewanella* strain. Strains were isolated from red (*Haliotis rufescens*) and white (*Haliotis sorenseni*) abalone, with and without exposure to “*Candidatus* Xenohaliotis californiensis,” the causative agent of abalone withering syndrome.

## GENOME ANNOUNCEMENT

Withering abalone syndrome (“*Candidatus* Xenohaliotis californiensis” infection) has caused a large decline in the population of abalone in coastal California in recent years (1). In this study, we isolated bacteria from the feces of both red abalone (*Haliotis rufescens*) and white abalone (*Haliotis sorenseni*) with and without exposure to “*Ca. Xenohaliotis californiensis*.” All of the resulting strains for which we obtained genome sequence data were either *Vibrio* or *Shewanella* species. *Vibrio* is a genus of Gram-negative marine bacteria that can cause illness (e.g., cholera and vibriosis) in humans and animals. *Shewanella* species are normal flora of shellfish and are not known to cause disease.

Abalone feces was streaked onto seawater agar (15.0 g of agar, 5.0 g of peptone, 2.0 g of beef extract, 0.5 g of KNO_3_, and 1.0 liter of InstantOcean), Columbia blood agar, lysogeny broth (LB), and Difco seawater medium. Liquid cultures were prepared from single colonies and grown at room temperature for four days. DNA was isolated using a Qiagen DNeasy blood and tissue kit. A 16S rRNA gene product was amplified using the 1391R (5′-GACGGGCGGTGTGTRCA-3′) and 27F (5′-AGAGTTTGATCMTGGCTCAG-3′) universal primers. Isolates were identified by Sanger sequencing of the PCR product. Sequencing libraries were constructed using a Kapa HyperPlus kit, and libraries were size selected to 600 to 900 bp using a BluePippin platform (Sage Science). Paired-end (PE) 300-bp sequencing was performed on an Illumina MiSeq platform.

An average of 682,098 reads were generated for each of the *Vibrio* strains, and 534,102 reads were generated for the *Shewanella* strain (Table 1). All sequence processing and assembly was performed using the A5-miseq assembly pipeline (version 20150522). This pipeline automates the processes of data cleaning, error correction, contig assembly, and quality control (2, 3).

**TABLE 1:** Genome assembly information

Strain	Accession no.	Host species	WS exposure^a^	No. of contigs	Genome size (bp)	*N*_50_ (bp)	No. of raw reads	Coverage (×)	No. of genes	No. of RNAs
*Vibrio cyclitrophicus* UCD-FRSSP16_1	LZFR00000000	*H. rufescens*	Exposed	66	5,051,153	373,940	821,306	49	4,362	198
*Vibrio cyclitrophicus* UCD-FRSSP16_8	LZFZ00000000	*H. sorenseni*	Exposed	64	5,018,558	550,710	722,502	43	4,351	199
*Vibrio* sp. UCD-FRSSP16_10	LZFX00000000	*H. rufescens*	Exposed	81	3,599,647	147,192	717,028	60	3,168	155
*Vibrio splendidus* UCD-FRSSP16_15	LZGA00000000	*H. rufescens*	Unexposed	44	5,379,662	819,026	577,438	32	4,658	179
*Vibrio cyclitrophicus* UCD-FRSSP16_18	LZFT00000000	*H. sorenseni*	Unexposed	50	5,046,131	534,326	710,666	42	4,394	184
*Vibrio tasmaniensis* UCD-FRSSP16_25	LZFS00000000	Unknown	Unknown	39	5,556,487	968,710	643,116	35	4,827	175
*Vibrio* sp. UCD-FRSSP16_30	LZFW00000000	*H. rufescens*	Exposed	85	3,606,693	175,784	667,338	56	3,167	151
*Vibrio cyclitrophicus* UCD-FRSSP16_31	LZFU00000000	Unknown	Unknown	94	4,963,458	495,080	640,182	39	4,330	192
*Vibrio tasmaniensis* UCD-FRSSP16_35	LZFY00000000	*H. sorenseni*	Exposed	73	5,660,313	390,830	778,512	41	4,963	180
Vibrio averages				66	4,853,869	510,207	682,098	44	4,232	177
*Shewanella* sp. UCD-FRSSP16_17	LZFV00000000	*H. sorenseni*	Unexposed	51	4,965,867	603,668	534,102	33	4,319	125

^a^ WS, Withering Syndrome.

The final *Vibrio* assemblies had an average of 66 contigs, with an average genome size of 4.85 Mbp and an assembly *N*_50_ of 510,207 bp (Table 1). The assembly for *Shewanella* sp. strain UCD-FRSSP16_17 contained 51 contigs, a genome size of 5 Mbp, and an *N*_50_ of 603,668 bp. Completeness of the genomes was assessed using the PhyloSift software (4), which searches for a list of 37 highly conserved single-copy marker genes (5), of which all 37 were found in all assemblies.

Automated annotation was performed using the RAST annotation server (6). *Shewanella* sp. UCD-FRSSP16_17 contains an estimated 4,319 protein-coding sequences and 125 noncoding RNA sequences. The *Vibrio* isolates contain an estimated average 4,232 protein-coding sequences and 177 noncoding RNA sequences (Table 1).

Taxonomy was determined for *Shewanella* sp. UCD-FRSSP16_17 by taking the full-length 16S rRNA sequence from RAST, adding to an alignment of *Shewanella* strains at the Ribosomal Database Project (RDP) (7), and inferring a maximum-likelihood tree with FastTree (8). Because the resulting tree contained polyphyletic clades and significant ambiguity, we did not assign a species name to this isolate. For all *Vibrio* strains, we generated a whole-genome concatenated marker tree. This tree was inferred from an alignment of 441 *Vibrio* genomes and contained mostly well-supported monophyletic clades that allowed us to assign species names to the *V. cyclitrophicus*, *V. splendidus*, and *V. tasmaniensis* isolates.

### Accession number(s).

All 10 assemblies described in this paper have been deposited as whole-genome shotgun projects in DDBJ/EMBL/GenBank under the accession numbers provided in Table 1.
